# S03 A physical literacy eco-system in Denmark: from research to practice … and back again

**DOI:** 10.1093/eurpub/ckac093.011

**Published:** 2022-08-29

**Authors:** Peter Elsborg, Peter Bentsen, Glen Nielsen, Paulina Sander Melby, Knud Ryom, Mette Kurtzhals

**Affiliations:** 1 Center for Clinical Research and Prevention, Copenhagen University Hospital – Bisbebjerg and Frederiksberg, Frederiksberg, Denmark; 2 Department of Nutrition, Exercise and Sports, University of Copenhagen, Copenhagen, Denmark; 3 Department of Public Health, Applied Public Health Research, Aarhus University, Aarhus, Denmark; 4 Department of Geosciences and Natural Resource Management, University of Copenhagen, Copenhagen, Denmark

**Keywords:** Interventions, life long participation, physical Literacy, research

## Abstract

The burden of non communicable diseases is a rising global problem and the increasing inactivity seen all over the world is extremely costly to societies (Ding et al., 2016). A recent Danish evaluation on physical activity (PA) showed that a considerable number of Danish children and adolescents do not meet national PA recommendations (The Ministry of Health, 2019). Viewing this problem through a life course perspective points to the solution of making sustainable behavioural changes with children (Frohlich & Potvin, 2010). Across the life span, there is clear evidence of the health-enhancing benefits of PA (Lee et al., 2012). However, at the same time, ample empirical evidence supports that extrinsic motives for participating in PA, such as increasing health, are insufficient when long term participation is the goal. This means that prevention and health promotion interventions should not blindly target the factors and behaviours that predict adult health, such as PA, but move towards intervening on the ‘so-called’ causes of the causes such as factors enabling a physical active lifestyle throughout life. Physical literacy (PL) is a comprehensive multidimensional concept that has been identified as a ‘cause of the causes’. PL consists of dimensions that lay the foundation for an individual’s capacity and tendency for engaging in physical activities throughout life (Whitehead, 2001). In this symposium, we aim to present four research and interventions projects from the education, sport and public health sectors that have emerged in the last couple of years, and through the presentation of these projects, how we in Denmark have established a PL ecosystem between research and practice – and how our national collaboration, PL-net, has contributed to this.

**Program:**

1) Introduction to the symposium

2) Physical literacy, physical activity and wellbeing in Danish children

3) The Child-COOP Denmark study: using physical literacy to guide and evaluate a systemic approach to health promotion

4) Staying active together in sports: the importance of and how to promote the emotional domain of physical literacy

5) 3PL: Promoting pupils’ physical literacy: a pilot study testing feasibility and acceptability of the Y-PATH intervention in a Danish school setting

6) The symposium will be ended by a panel debate where it also will be possible for the participants to comment and ask questions.

